# Anti-EpCAM Functionalized I-131 Radiolabeled Biomimetic Nanocarrier Sodium/Iodide-Symporter-Mediated Breast-Cancer Treatment

**DOI:** 10.3390/bioengineering9070294

**Published:** 2022-06-30

**Authors:** Suphalak Khamruang Marshall, Yada Panrak, Naritsara Makchuchit, Passara Jaroenpakdee, Boonyisa Saelim, Maneerat Taweesap, Verachai Pachana

**Affiliations:** 1 Department of Radiology, Faculty of Medicine, Prince of Songkla University, Songkhla 90110, Thailand; panrak.yp@gmail.com (Y.P.); makchuchit.nm@gmail.com (N.M.); jaroenpakdee.pj@gmail.com (P.J.); saelim.slbys@gmail.com (B.S.); taweesap.mt@gmail.com (M.T.); pachana.vp@gmail.com (V.P.); 2 Department of Biomedical Sciences and Biomedical Engineering, Faculty of Medicine, Prince of Songkla University, Songkhla 90110, Thailand

**Keywords:** breast cancer, drug delivery, EpCAM, I-131, ionizing radiation, nanoparticle, PLGA

## Abstract

Currently, breast-cancer treatment has a number of adverse side effects and is associated with poor rates of progression-free survival. Therefore, a radiolabeled anti-EpCAM targeted biomimetic coated nanocarrier (EINP) was developed in this study to overcome some of the treatment challenges. The double emulsion method synthesized the poly(lactic-co-glycolic acid) (PLGA) nanoparticle with Na^131^I entrapped in the core. The PLGA nanoparticle was coated in human red blood cell membranes and labeled with epithelial cell adhesion molecule (EpCAM) antibody to enable it to target EpCAM overexpression by breast-cancer cells. Characterization determined the EINP size as 295 nm, zeta potential as −35.9 mV, and polydispersity as 0.297. EINP radiochemical purity was >95%. Results determined the EINP efficacy against EpCAM positive MCF-7 breast cancer at 24, 48, and 72 h were 69.11%, 77.84%, and 74.6%, respectively, demonstrating that the EINPs achieved greater cytotoxic efficacy supported by NIS-mediated Na^131^I uptake than the non-targeted ^131^INPs and Na^131^I. In comparison, fibroblast (EpCAM negative) treated with EINPs had significantly lower cytotoxicity than Na^131^I and ^131^INPs (*p* < 0.05). Flow cytometry fluorescence imaging visually signified delivery by EINPs specifically to breast-cancer cells as a result of anti-EpCAM targeting. Additionally, the EINP had a favorable safety profile, as determined by hemolysis.

## 1. Introduction

According to GLOBCAN 2020 statistics, breast cancer is the most common cancer in women worldwide, with 2,261,419 new cases and 684,996 mortalities yearly. The severity of breast cancer depends on the stage of disease, tumor size, and extent of any metastases. Treatment methods for breast cancer include surgery, radiation therapy, chemotherapy, hormone therapy, and targeted therapy [1]. Furthermore, for women diagnosed with non-metastatic invasive breast cancer, the average 10-year survival rate is 84%. However, if the invasive breast cancer is solely found in the breast, the 5-year survival rate rises to 99 percent, and 65 percent of women with breast cancer are diagnosed at this stage [2].

In nuclear medicine, the radiopharmaceutical ^131^I has been the principal diagnostic and therapeutic agent for thyroid cancer and the first theranostic agent, as it emits both 90% beta particles (therapeutic) to destroy cancer cells and 10% gamma particles (diagnostic) [3]. Currently, ^131^I is mainly used for thyroid treatment because the thyroid gland has a sodium-iodide symporter (NIS), a glycosylate-containing integral membrane protein that mediates intracellular iodine transport and regulates thyroid ^131^I accumulation [3]. Additionally, NIS is also expressed in mammary glands, the ciliary body of the eye, the choroid plexus, the stomach parietal cells, and salivary glands. Consequently, prostate cancer prostate-specific antigen (PSA) NIS expression was one of the early NIS investigations, which established that NIS expression resulted in specific uptake of ^131^I and the death of androgen-sensitive human prostate adenocarcinoma (LNCaP) cells [4]. As a result, there has been increased research evaluating the application of ^131^I to treat other cancers [5,6,7,8]. Moreover, Wapnir, Irene L., et al. assessed the NIS-mediated I^-^ accumulation in breast-cancer metastases, confirming that metastatic sites accumulated iodide [9]. Additionally, a study by Chatterjee, Sushmita, et al. determined that NIS expression was detected in 70% of breast-cancer patients, and NIS activity varied amongst breast-cancer subtypes [10].

Despite advances in breast-cancer treatment, chemotherapy medications have been the gold standard for cancer treatment; however, they can adversely affect the patient’s immune system [11]. Moreover, a large percentage of chemotherapeutic agents have a short half-life and a high clearance rate in circulation. In addition, most medications used in clinical practice are low-molecular-weight compounds that disperse uniformly throughout the body and rapidly diffuse into healthy tissues, resulting in only trace quantities of the medicine reaching the target site [12]. However, cancer therapy has become more tailored to the particular type of cancer, along with new developments in medication delivery technologies. As a result, the variety of systems is growing, and some have shown promise in incorporating nanoparticles (NPs) into their applications. In particular, the delivery of pharmaceuticals into cells by targeted nanocarriers represents an alternative pathway for drug diffusion [13]. The need for nanocarriers that can overcome human biology has inspired the development of nanocarriers constructed from biological materials, such as red blood cells that have gained momentum [14]. Such biometric nanocarriers have demonstrated that coating nanoparticles with cell membranes increases circulation time and imparts added functionality [15]. Furthermore, CD47 and other RBC membrane proteins act as self-markers, preventing immune clearance and resulting in prolonged circulation. CD47 operates as a “don’t eat me” signal on the macrophage surface, inhibiting RBC macrophage engulfment [16]. For example, Hu, Che-Ming J., et al. ascertained that PEGylated PLGA NPs had a half-life of 15.8 h, while in contrast, RBCm-coated PLGA NPs had a significantly longer half-life of 39.6 h [17]. In addition, homophilic synaptic targeting was accomplished by coating dendritic mesoporous silica nanoparticles with the cancer cell membrane, achieving low cytotoxicity, good biocompatibility, and homotypic-targeting [18].

Additionally, a variety of targeting moieties, such as antibodies, aptamers, peptides, and small molecules, can be conjugated with nanoparticles to facilitate the specific binding of nanoparticle probes to targets of interest for cancer diagnostics and therapeutics [19]. For instance, epithelial cell adhesion molecule (EpCAM) is an excellent target for tumor diagnostics and treatment because of its tumor-specific overexpression in cancer tissues, such as adenocarcinomas in the colon, stomach, pancreas, and epithelial breast cancer [20]. In fact, Osta, Walid A., et al. determined that in primary and metastatic breast cancer, EpCAM was overexpressed by 100 to 1000 times [21].

However, an anti-EpCAM human RBCm (hRBCm)-coated radiolabeled PLGA NP has not yet been reported for MCF-7 (EpCAM+) targeted treatment. Hence, this proof-of-concept in vitro study recognizes the importance of developing a more efficacious targeted cancer treatment for breast cancer. Therefore, we developed a targeted theranostic biomimetic drug delivery platform using hRBCm-coated anti-EpCAM functionalized Na^131^I radiolabeled PLGA nanoparticles (EINPs) to treat MCF-7 breast-cancer cells.

## 2. Materials and Methods

### 2.1. Materials and Cell Culture

Human blood for this study was donated by the Faculty of Medicine, Prince of Songkla University, Thailand. MCF-7 breast-cancer cells (EpCAM+) and fibroblast (EpCAM-) were given by the Department of Biomedical Science and Biomedical Engineering, Faculty of Medicine, Prince of Songkla University. The Na^131^I was purchased from Thailand Institute of Nuclear Technology. Biotin anti-human CD326 (EpCAM) antibody and streptavidin-FITC were purchased from Biolegend (San Diego, CA, USA). 1,1′-dioctadecyl-3,3,3′,3′–tetramethylindodicarbocyanine 4-chlorobenzenesulfonate salt (DiD) was obtained from Sigma Aldrich (St. Louis, MO, USA). 1,2-distearoyl-sn-glycero-3-phosphoethanolamine-N-[biotin(polyethylene glycol)-2000] (ammonium salt) DSPE-PEG-(2000)-Biotin was supplied by Avanti Polar Lipids (Birmingham, AL, USA). NuPAGE^TM^ LDS sample buffer, Coomassie blue, anti-Human IgG secondary antibody, β-actin loading control monoclonal antibody, and SDS-polyacrylamide gel were procured from Thermo Fisher Scientific (Waltham, MA, USA). Dulbecco’s Modified Eagle Medium (DMEM) was purchased from Thermo Fisher Scientific (Waltham, MA, USA). Phosphate buffer solution (PBS) was supplied by Thermo Fisher Scientific (Waltham, MA, USA). Carboxy-terminated 50:50 PLGA polymer 0.66 dL/g was obtained from LACTEL Absorbable Polymers (Birmingham, AL, USA). The bicinchoninic acid (BCA) assay kit and paraformaldehyde were acquired from Millipore Sigma (St. Louis, MO, USA). The MTT assay kit (cell proliferation) (colorimetric) was acquired from Abcam (Cambridge, MA, USA). Purified deionized (DI) water provided by a Direct-Q3 water purification system was used to prepare all aqueous solutions. Millipore Sigma (St. Louis, MO, USA) and Thermo Fisher Scientific (Waltham, MA, USA) supplied the solvents used in the research. The Live/Dead™ cell imaging kit (488/570) was acquired from Thermo Fisher Scientific (Waltham, MA, USA). The CellTiter-Glo^®^ 3D Cell Viability Assay kit purchased from Promega Corporation (Madison, WI, USA). Instant thin-layer chromatography medium (ITLC-SG) papers were purchased from Global Medical Solutions, Thailand. Chloroform-methanol with 0.25% KCl solution was supplied by Thermo Fisher Scientific (Waltham, MA, USA). The CRC^®^-77tHR Dose Calibrator was supplied by Capintec, Inc. (Florham Park, NJ, USA).

### 2.2. Preparation and Characterization of Nanoparticles

In this study, cell culture medium Dulbecco’s Modified Eagle Medium (DMEM) was used as control. The PLGA nanoparticles were prepared by the nanoprecipitation method. Briefly, PLGA was dissolved in dichloromethane organic solvent (DCM), the PLGA was added dropwise to 1× PBS aqueous solution and stirred gently for 4 h to assist evaporation in a fume hood. Sodium iodide (Na^131^I) 925 MBq (25 mCi) was dissolved in 1× PBS.

In the next phase, Na^131^I radiolabeled PLGA nanoparticles were synthesized using the double emulsion water-in-oil-in-water (w/o/w) solvent evaporation process. 1,1′-dioctadecyl-3,3,3′,3′-tetramethylindodicarbocyanine, 4-chlorobenzenesulfonate salt (DiD) was incorporated into a PLGA nanoparticle core during the oil phase to enable validation of cellular uptake. Na^131^I activity 925 MBq (25 mCi) was dissolved in 1000 μL of 1× PBS as the inner phase, in and 5000 μL of 10 mg/mL PLGA in DCM as the organic phase, then emulsification was performed by sonication at 70% pulsed power (2 s on/1 s off) for 2 min to create a water-in-oil solution. Additionally, 5000 μL of 1× PBS as the water phase was then added to the solution and sonicated for 2 min. Afterward, the solution was mixed with 10 mL of 1× PBS and gently stirred for 4 h in a fume hood to promote evaporation at room temperature in order to create the water-in-oil-in-water solution.

Furthermore, to produce human red blood cell membrane (hRBCm) vesicles, human red blood cells (RBCs) were isolated from whole blood by centrifugation at 15,000 rpm (24,016× *g*) for 10 min at a temperature of 4 °C to remove the plasma and buffy coating. Next, RBCs were washed with 1× PBS, 0.25× PBS was added for hypotonic treatment for 5 replicates, and hemoglobin was separated by centrifugation for 5 min at 800× *g*. On completion, the hRBCm pellets were collected and stabilized in 1× PBS to remove the plasma and buffy coating at a temperature of 4 °C.

Additionally, to fabricate anti-EpCAM functionalized hRBCm, 500 µL of hRBCm was suspended in 4 mL 1× PBS and incubated in 0.5 mg DSPE-PEG-biotin (3700 D, 100 g/mL) at room temperature, then stirred at 400× *g* for 30 min. The mixture was then washed twice with 1× PBS, stirred at 400× *g* for 5 min, and suspended in 1× PBS. Next, 1 µg of streptavidin-FITC solution (6.30 × 10^4^ molecules/RBC) was added and stirred gently at 4 °C for 60 min. Afterward, the hRBCm-biotin-streptavidin was washed with 1× PBS to remove any streptavidin residue and stirred for 5 min at 400× *g*. In addition, the biotin anti-human CD326 (EpCAM) antibody and hRBCm-biotin-streptavidin were mixed at 4 °C and continuously stirred for 60 min; then the EpCAM antibody-hRBCm was washed twice with 1× PBS and stored at 4 °C. Finally, the Na^131^I radiolabeled PLGA NPs 10 mg/mL were mixed with hRBCm vesicles and functionalized anti-EpCAM to coat the nanocarrier. The PLGA and hRBCm ratio was 1:0.5. For each batch, 5 mg of PLGA NPs and 2.5 mg of hRBCm vesicles were used. Then, before coating onto the PLGA cores, the hRBCm vesicles were functionalized with anti-EpCAM antibodies, and the following procedure was used per batch:

To functionalize the 2.5 mg of hRBCm vesicles with anti-EpCAM, biotinylated anti-EpCAM 1 μg (2.5 × 10^−11^ mol, MW = 40 kg/mol) was added to the hRBCm. Therefore, 1.5 × 10^13^ molecules of biotinylated anti-EpCAM was added to 5 mg of PLGA NPs. As a result, anti-EpCAM 3 × 10^12^ molecules/1 mg PLGA NPs was used in this platform. A nanoparticle tracking analyzer (NTA) (NanoSight, Malvern analytical) established that each 1 mg of PLGA contained ~9 × 10^10^ particles. Therefore, the quantity of anti-EpCAM per nanoparticle was ~33.

**Figure 1:**
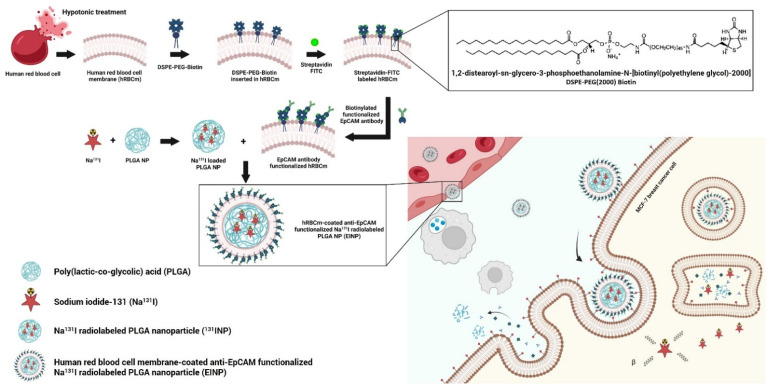
The fabrication of EINPs. Preparation of red blood cell membrane-derived vesicles: modification of human red blood cell membranes (hRBCm) with DSPE-PEG-Biotin, conjugation of streptavidin-FITC with biotin, and addition of biotin-functionalized anti-EpCAM to streptavidin. Nanoparticle preparation involves encapsulation of the Na^131^I into the nanoparticle and the coating of the nanoparticle with the hRBCm-derived vesicles.

In brief, the different nanoformulations were as follows (Figure 1):

- Control: Dulbecco’s Modified Eagle Medium (DMEM) cell culture medium.
- PLGA: PLGA nanoparticle pellets were dissolved in dichloromethane organic solvent (DCM), added to 1× PBS, and agitated gently for 4 h to aid evaporation.
- Na^131^I: sodium iodide (Na^131^I) with an activity of 925 MBq (25 mCi) was dissolved in 1× PBS.
- ^131^INPs: Na^131^I with an activity of 925 MBq was loaded into the PLGA cores during the inner phase.
- EINPs: To fabricate the targeted EINPs, the ^131^INPs were functionalized with a streptavidin-FITC and biotin anti-human CD326 (EpCAM) antibody-targeting ligand.

Furthermore, to evaluate EINP physiochemical properties, the EINPs were characterized for size, polydispersity, and zeta potential using dynamic light scattering (DLS) measurements (Malvern ZEN 3600 Zetasizer, Westborough, MA, USA) in triplicate at room temperature. Additionally, to determine EpCAM antibody binding to the Na^131^I radiolabeled PLGA nanoparticles and MCF-7 uptake, streptavidin-fluorescein isothiocyanate (FITC) was used and the fluorescent intensity measured by a fluorescent live-cell imaging microscope (Lionheart FX Automated Microscope, BioTek Instrument, Santa Clara, CA, USA). Furthermore, a JEOL JEM-2010 transmission electron microscope (TEM) analyzed the EINP morphology, which was coated with 1 mg/mL of EINPs, placed on a TEM glow-discharged carbon-coated grid, and washed for 5 min with 10 drops of distilled water. It was then stained with 1% uranyl acetate, and the grid was dried and photographed using a TEM set to 200 kV.

### 2.3. Membrane Protein Analysis of EINPs

Sodium dodecyl sulfate-polyacrylamide gel electrophoresis (SDS-PAGE) was used to analyze both the self-recognition protein CD47, which has an immunomodulatory function [22], and CD326 (EpCAM) to facilitate EINP membrane protein analysis in comparison to hRBCm proteins. Briefly, EINPs and hRBCm were each produced in an SDS sample buffer, and the protein concentration quantified with a BCA assay kit. The protein samples were denatured on a heat block, and samples centrifuged (1,400 rpm for 1 min). The EINP and hRBCm samples were then loaded into cells containing SDS-polyacrylamide gel, stained with Coomassie blue, and imaged. Briefly, western blot analysis was performed to detect the CD47 and CD326 proteins of interest by transferring the gel onto the membrane. The samples were prepared using NuPAGE^TM^ LDS sample buffer (Thermo Fisher Scientific (Waltham, MA, USA)), separated by SDS-PAGE, relocated to the nitrocellulose membranes, and treated overnight with primary antibodies CD47 and CD326, and with β-actin loading control monoclonal antibody. They were then conjugated with secondary antibodies anti-human IgG peroxidase, enabling the proteins to be detected by Immobilon^TM^ Western Chemiluminescent HRP Substrate. 

### 2.4. Radiochemical Purity and Radioactive Stability of EINPs

Furthermore, the standard radiochemical purity percentage (%RCP) was determined by a three-paper chromatography strip and solvent system, which involved dropping 100 µL of EINPs onto the instant thin-layer chromatography (ITLC-SG) at 1 drop/sheet. The ITLC-SG paper was developed with chloroform-methanol and 0.25% KCl solution. Moreover, the unbound I-131 and EINPs were determined using a dose calibrator in which only unbound I-131 migrates to the top of the strip. Spots of radioactivity were identified according to their R_f_ value. Unbound I-131 migrated with the solvent front at R_f_ = 0.6–1.0, while EINPs migrated at R_f_ = 0.0. Impurities should not exceed 5% of overall activity. The formula for calculating the percent radiochemical purity of EINPs is as follows:

(1) $$\%\mathrm{RCP}\;=\frac{\mathrm{Total}\;\mathrm{counts}\;\mathrm{of}\;\mathrm{sample}\;}{\mathrm{Total}\;\mathrm{counts}\;\mathrm{of}\;\mathrm{sample}\;+\mathrm{unbound}\;I-131}\times 100$$

The ^131^INPs and EINPs were incubated for 0, 1, 3, 6, 12, and 24 h in 20 percent fetal bovine serum and 1× PBS at 37 °C. Using a 0.9% NaCl solution as the eluent, ITLC-SG was used to determine the stabilities of six serial serum samples. Over time, the percentage change in the radiochemical purity of I-131-labeled nanoparticles dictated their stability. The same processes were carried out and evaluated in triplicate.

### 2.5. Calculation of the Amount of Na^131^I Radiation Delivered to Each Well

The dosage per well of radiation for Na^131^I activity 3.70 MBq (100 μCi) was determined using the following formula:

(2) $$D\;=\;Ã\;\times \frac{\Delta \beta }{100}$$

where D is the absorbed dose of radiation, Ã is the cumulative activity, and Δ β is β rays absorbed dose constant.

(3) $$Ã\;=\overset{t}{\underset{0}{\int }}A\left(t\right)\;d\left(t\right)$$

(4) $$A\left(t\right)={\;A}_{0}{\;e}^{-\lambda t}$$

(5) $$\;Ã\;=\frac{A_{0}\left[\;\left(1-e^{-\lambda t}\right)\right]}{\lambda }$$

where A(t) is the activity at time t, A_0_ is the initial activity at t = 0, and λ is the decay constant.

### 2.6. Two-Dimensional (2D) In Vitro Therapeutic I-131 Dose Optimization of EINPs

In vitro therapeutic response was determined using the MTT assay to determine the cell proliferation rate. MCF-7 (EpCAM+) breast-cancer cells were cultured in Dulbecco’s Modified Eagle Medium (DMEM) supplemented with 1% penicillin/streptomycin, 10% fetal bovine albumin, and L-glutamine (Gibco-BRL). MCF-7 cells were cultivated to 60–80% confluency in culture flasks at 37 °C and 5% CO_2_ in a humidified incubator. Twenty-four hours before treatment, 5000 cells per well were plated onto 96-well microtiter plates with a final volume of 200 μL for each well. The MCF-7 cells were then treated with EINPs (n = 3) at doses of 0, 0.37, 1.85, 3.70, 5.55, and 7.40 MBq for 24 h. The EINPs was washed and cultivated in the new culture mix for 24 h at a final volume of 200 μL/well. Following that, the cell proliferation rate was determined using the MTT test, incubating 50 μL serum-free medium with 50 μL MTT solution in 96-well microtiter plates for 3 h at 37 °C and 5% CO_2_. After adding 150 μL MTT solvent to each well, the well plates were covered with aluminum foil to avoid light penetration and stirred for 15 min in an orbital shaker. A microplate reader set to optical density (OD) = 590 nm was used to determine the absorbance of the treated cells. The color intensity generated was related to the number of viable cells. For each dosage, the following cell proliferation rate percentage (P) was calculated:

(6) $$P\;\left(\%\right)=\frac{\mathrm{ODc}\;-\;\mathrm{ODs}}{\mathrm{ODc}}\times 100$$

where P (%) is the cell proliferation rate percentage, ODc is mean optical density of control, and ODs is the sample’s mean optical density. Proliferation rate percentage is reported as a percentage based on the mean of three measurements. Furthermore, the greater the metabolic activity of the MCF-7 cells, the greater the signal detected indicating cell proliferation, cell viability, and cytotoxicity.

### 2.7. Radioactive Encapsulation Efficiency

An amount of 3.70 MBq of I-131 in NPs was extracted using 0.1 M HCl in acetonitrile solution to test the encapsulation efficiency of the EINPs at various time periods. The concentration of I-131 was identified by determining its activity using a gamma counter. The encapsulation efficiencies were computed using Equations (5) and (7) for radioactive decay at various time intervals. All experiments were conducted in triplicate, and the findings are shown as the average of the three values (mean ± standard deviation; n = 3).

(7) $$\mathrm{Encapsulation}\;\mathrm{efficiency}\;\left(\%\right)=\frac{\mathrm{total}\;\mathrm{Iodine}\;131\;\mathrm{activity}\;\mathrm{added}\;-\;\mathrm{not}\;\mathrm{encapsulated}\;\mathrm{Iodine}\;131}{\mathrm{total}\;\mathrm{Iodine}\;131\;\mathrm{activity}\;\mathrm{added}}\;\times \;100$$

### 2.8. Two-Dimensional (2D) In Vitro Therapeutic Response and Efficacies

The MCF-7 cells were cultured in Dulbecco’s Modified Eagle Medium (DMEM) and augmented with 10% fetal bovine albumin, 1% penicillin/streptomycin, and L-glutamine. All the MCF-7 cells were cultured in culture flasks to 60–80% confluency at 5% CO_2_ and 37 °C in a humidified incubator. The cells were plated in 96-well microtiter plates at 5000 cells/well with final volumes of 200 µL/well for 24 h before treatment. Afterward, the MCF-7 cells (n = 3) were treated with control (DMEM), PLGA, Na^131^I activity 3.70 MBq (100 µCi) per well, ^131^INPs activity 3.70 MBq per well, and EINPs activity 3.70 MBq per well for 24, 48, and 72 h. After the treatments, the control, PLGA, Na^131^I, ^131^INPs, and EINPs were washed and incubated for 24 h at 200 µL/well final volumes in fresh culture media. 

The MTT viability was performed by mixing 50 µL serum-free medium and 50 µL MTT solution in 96-well microtiter plates and incubating at 37 °C for 3 h. Subsequently, 150 µL of MTT solvent was added to each well, and the well plates were covered with aluminum foil to prevent light ingress and shaken for 15 min in an orbital shaker. A microplate reader determined the treated cells’ absorbance at OD = 590 nm.

### 2.9. In Vitro Targeting Efficiency upon Cellular Binding/Uptake and Surface Immunofluorescence

The intracellular binding/uptake of both the MCF-7 (EpCAM+) and fibroblast (EpCAM-) cells (n = 3) was established by treating the cells with EINPs activity 3.70 MBq at 200 µL/well final volumes, then incubated for 24 h. This was followed by washing the MCF-7 and fibroblast cells with 1× PBS and cells fixed with 4% paraformaldehyde for 20 min to preserve the cell samples, then washing with 1× PBS. The cells were then incubated for 30 min at 37 °C with 4′,6-diamidino-2-phenylindole (DAPI) nuclear staining dye and washed twice before fluorescent live-cell imaging microscope analysis. 

For nanoparticle surface immunofluorescence and cellular binding/uptake, flow cytometry was performed using ImageStreamX Mk II, and the MCF-7 cells were cultured in culture flasks to 80% confluency with 5% CO_2_ at 37 °C in a humidified incubator. Afterward, the cells were dissociated with 0.25% Trypsin-EDTA solution from the culture flasks. Furthermore, the cells were then centrifuged at 4000 rpm for 3 min to collect 1,000,000 cells, and the supernatant was washed twice at 4 °C with 1× PBS. Next, a single-cell suspension, MCF-7 (n = 3), was treated with control, PLGA, Na^131^I activity 3.70 MBq, ^131^INPs activity 3.70 MBq, and EINPs activity 3.70 MBq for 1 h. This was followed by washing the cells with 2 mL of 1× PBS, centrifuging at 350× *g* for 5 min, and washing again. The cells were suspended in 1× PBS buffer and flow cytometer analysis was performed. Flow cytometer cellular fluorescence was determined in bright-field (grey channel), DiD (red channel), and EpCAM (FITC; green channel) for a minimum of 50,000 cells/sample.

### 2.10. Two-Dimensional (2D) In Vitro Live/Dead upon Cellular Binding/Uptake of EINPs

The MCF-7 and fibroblast cells (n = 3) were treated with control, PLGA, Na^131^I activity 3.70 MBq, ^131^INPs activity 3.70 MBq, and EINPs activity 3.70 MBq at 72 h. After 72 h, the cell culture media were removed and the cells were washed with 1× PBS. Next, the Live Green (Comp. A) 1 mL (1 μM solution) was mixed with the Dead Red (Comp. B) 1 μL to create a 2× working solution. This was followed by the addition of 50 µL of the Live Green and Dead Red mixture to each well in equal volumes of 2× working solution. These were incubated for 30 min at 37 °C prior to fluorescent live cell imaging microscope analysis.

### 2.11. Three-Dimensional (3D) Human Tumor Spheroid In Vitro Live/Dead Cell Imaging

The MCF-7 cell viability was visualized following EINP treatment using a two-color fluorescence live/dead cell-imaging kit (488/570) (ThermoFisher Scientific, Waltham, MA, USA). MCF-7 cells were grown in a DMEM medium supplemented with 10% fetal bovine albumin, 1% L-glutamine, and 1% penicillin/streptomycin. The MCF-7 spheroids were synthesized as formerly described [23]. MCF-7 cells were grown to 60–80% confluence in a humidified 37 °C incubator with 5% CO_2_. Next, 5000 MCF-7 cells/well were seeded in 96-well ultra-low attachment multiple well plates for 72 h before treatment, with a final volume of 200 µL/well. MCF-7 cell-derived spheroids were treated with PLGA, Na^131^I, ^131^INPs, and EINPs at an equivalent I-131 activity of 3.70 MBq (100 μCi) for 72 h before being rinsed with 1× PBS. The untreated group served as the negative control, and each experiment was conducted three times. Next, two-color fluorescence live/dead cell imaging was prepared according to the manufacturer’s instructions. First, Live Green (Comp. A) was added to Dead Red (Comp. B) and blended to make a 2× solution. After adding 50 μL of 2× working solution to each well, they were incubated for 15 min at 25 °C, and Lion Heart live cell imaging was performed to image the cells.

A 3D CellTiter-Glo cell viability assay was used to determine the MCF-7 spheroid cell viability after treatment with PLGA, Na^131^I, ^131^INPs, and EINPs at an I-131 equivalent activity of 3.70 MBq (100 μCi) for 72 h. Following the manufacturer’s instructions, the medium was removed, and 100 µL of CellTiter-Glo reagent was added to each well, mixed for 2 min, and then incubated at room temperature for 30 min. The luminescence was then determined using a plate reader (Tecan Infinite 200 PRO, Männedorf, Switzerland).

### 2.12. EINPs Penetration in Three-Dimensional (3D) Human Tumor Spheroid

The penetration of EINPs into the tumor spheroids was studied using a 3D MCF-7 human spheroid model, and spherical MCF-7 aggregates formed. One spheroid per well was exposed to EINPs with an activity of 3.70 MBq (100 μCi) for 24, 48, and 72 h, and the untreated group was used as a negative control. The wells were rinsed with 1× PBS and then fixed in 1× PBS with 4% paraformaldehyde for 60 min. Then the spheroids were washed and stained with DAPI and scanned using Lion Heart live cell imaging, and the fluorescence intensities of EINPs were calculated.

### 2.13. Analysis of Nanoparticle Hemolytic Properties In Vitro: Blood Compatibility

The human blood was collected in the presence of a heparin anticoagulant to prevent blood clot formation, and the plasma removed. Next, the RBCs were suspended at 8 × 10^9^ cells/mL in the positive control (DMSO), Na^131^I activity 3.70 MBq, ^131^INPs activity 3.70 MBq, and EINPs activity 3.70 MBq, and centrifuged at 500× *g* for 5 min. The supernatants were collected at 0, 3, 6, 12, 24, 36, 48, and 80 h at 37 °C to determine blood lysis. A BCA protein assay kit was used to quantify the total protein in the supernatant samples prior to hemoglobin absorbance measured by a microplate reader at OD = 562 nm to evaluate hemolysis percentage.

### 2.14. Statistical Analysis

All investigations in this research were conducted in triplicate (both experimentally and analytically), and all findings and experimental data are presented as mean ± SD. All statistical analyses were conducted using GraphPad Prism 8.0 (GraphPad Software Inc., San Diego, CA, USA). The unpaired, two-tailed Student’s *t*-test conducted pairwise statistical comparisons. Additionally, one-way ANOVA was performed to compare the means of two or more independent groups to determine if the means were statistically different, with *p* < 0.05 being statistically significant.

## 3. Results

### 3.1. Physicochemical Properties and Characterization of Nanoparticles

This study demonstrated that a biomimetic novel anti-EpCAM functionalized theranostic hRBCm-coated sodium iodide-131 (Na^131^I) radiolabeled PLGA nanoparticle (EINP) platform surmounted a number of the difficulties associated with drug delivery systems. Furthermore, this novel biomimetic nanocarrier successfully targeted and achieved cellular binding/uptake by MCF-7 breast-cancer cells. As seen in Figure 1, the EINPs were designed to primarily target cancer cells overexpressing EpCAM, entering the MCF-7 cells due to receptor-mediated endocytosis and eliminating them preferentially with Na^131^I.

**Figure 2:**
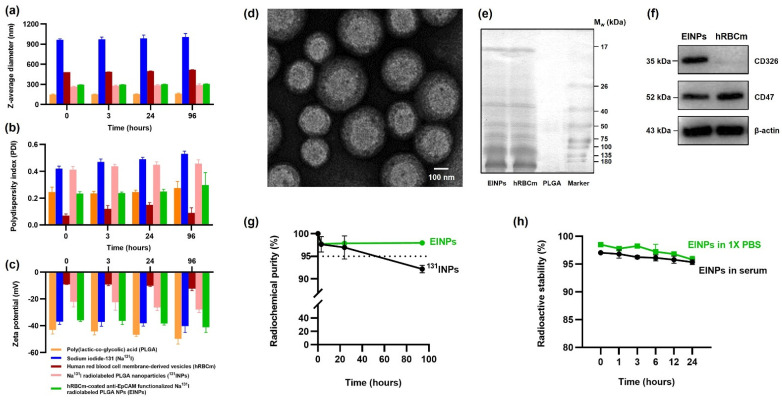
Physiochemical properties and characterization of PLGA, sodium iodide-131 (Na^131^I), human red blood cell membrane-derived vesicles (hRBCm), Na^131^I radiolabeled PLGA nanoparticles (^131^INPs), and hRBCm-coated anti-EpCAM functionalized Na^131^I radiolabeled PLGA NPs (EINPs). (**a**) Z-average diameter (nm) after 0, 3, 24, and 96 h incubation. (**b**) Polydispersity index (PDI) after 0, 3, 24, and 96 h incubation. (**c**) Zeta potential after 0, 3, 24 and 96 h incubation. (**d**) Transmission electron micrographs of EINPs (samples were stained negatively with uranyl acetate; scale bar = 100 nm). (**e**) EINPs membrane protein analysis by sodium dodecyl sulfate-polyacrylamide gel electrophoresis (SDS-PAGE). (**f**) Western blot EINPs and hRBCm protein analysis. (**g**) Radiochemical purity (RCP). (**h**) EINPs radioactive stability in 1× PBS and serum at 37 °C. [Bars show the mean ± SD (n = 3)].

The EINP physicochemical properties were evaluated by dynamic light scattering (DLS) measurement using a Malvern Zetasizer to investigate particle size, polydispersity index (PDI), and zeta potential. The results indicated an EINP z-average diameter of ~295 nm (Figure 2a), a PDI of 0.297 (Figure 2b), and a zeta potential of −35.9 mV (Figure 2c). The size of the PLGA core was ∼149 nm; after radiolabeling, the ^131^INP diameter was ~265 nm; and after coating with hRBCm, the EINP size was ~295 nm. After 96 h, the EINPs increased slightly in size to 308 nm, and a reduction was observed in zeta potential to −41.1 mV, suggesting the EINPs were stable. The EINP morphology is illustrated by TEM in Figure 2d. The translocation of proteins CD47 and CD326 (EpCAM) to the EINPs was verified by SDS-PAGE to confirm hRBCm coating (Figure 2e), and Western blotting confirmed the proteins on the EINPs (Figure 2f). The radiochemical purity (RCP) graph is shown in Figure 2g, illustrating that EINP RCP at 0, 3, 24, and 96 h was greater than 95%. The RCP was not significantly different from 0 to 96 h. Radioactive stability shown in Figure 2h demonstrates that the EINPs had good stability over 24 h.

### 3.2. Two-Dimensional (2D) In Vitro Therapeutic Response of EINPs

**Figure 3:**
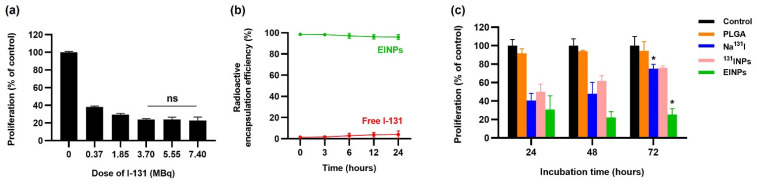
In vitro therapeutic responses of EINPs. (**a**) In vitro cytotoxicity using MTT assay of EINPs at dosages of 0, 0.37, 1.85, 3.70, 5.55, and 7.40 MBq after 24 h incubation. Denote ns = no statistically significant difference in efficacy of destroying cancer cells between 3.70, 5.55, and 7.40 MBq (*p* > 0.05). (**b**) Radioactive encapsulation efficiency after 0, 3, 6, 12, and 24 h incubation. (**c**) In vitro proliferation (% of control) using MTT assay of EINPs compared to control, PLGA, Na^131^I, and ^131^INPs treatment of MCF-7 breast-cancer cells, after 24, 48 and 72 h incubation. [Results represent mean ± SD (n = 3)]. * the significance between Na^131^I and EINPs at 72 h incubation (*p* < 0.05).

The graph (Figure 3a) describes the cell proliferation rate after various EINP radiation dose levels on MCF-7 cells using the MTT assay to determine the optimal radiation dosage for the cytotoxicity test. MCF-7 cells were treated with EINPs at dosages of 0, 0.37, 1.85, 3.70, 5.55, and 7.40 MBq. Increased apoptosis was reported at dosages of 3.70, 5.55, and 7.40 MBq. Similarly, Hosseinimehr, Seyed Jalal, et al. found that a dosage of 3.70 MBq generated an 8.5-fold increase in genotoxicity [24]. The MTT assay determined that a radiation dose of 3.70 MBq had a cell proliferation rate of 24%, a radiation dose of 5.55 MBq had a cell proliferation rate of 24%, and a radiation dose of 7.40 MBq had a cell proliferation rate of 26%. There was no statistically significant difference in the efficacy of destroying cancer cells. The EINP radioactive encapsulation efficiency was >95% over 24 h (Figure 3b).

In vitro proliferation analysis by MTT assay was performed to determine the therapeutic response efficacy, assessing the control, PLGA, Na^131^I, ^131^INPs, and EINPs. The cytotoxicity effect on MCF-7 cells was evaluated after incubation for 24, 48, and 72 h. As a result, Figure 3c indicates that the anti-EpCAM functionalized EINPs exerted more significant toxicity on the MCF-7 cells than Na^131^I and ^131^INPs. As illustrated at 24 h, the control proliferation rate was 100%, the EINPs cell proliferation rate was 30.89%, and the cell proliferation rate of Na^131^I was 40.54%. Furthermore, at 48 h, the EINPs mean cell proliferation rate was 22.16%. At 48 h, the Na^131^I cell proliferation rate was 47.96%, indicating Na^131^I and EINPs had a significant difference of *p* < 0.00001. Further, after 72 h, EINPs cell proliferation rate was 25.40%, with the control and EINPs having a significant difference of *p* < 0.00001. In addition, the cell proliferation rate of Na^131^I at 72 h was 74.99%, representing a significant difference between Na^131^I and EINPs. As shown in Figure 3c, the therapeutic response of EINPs resulted in a significant decrease in cell proliferation rate over the 72 h (*p* < 0.00001).

### 3.3. In Vitro Targeting Efficiency upon Cellular Binding/Uptake and Surface Immunofluorescence

**Figure 4:**
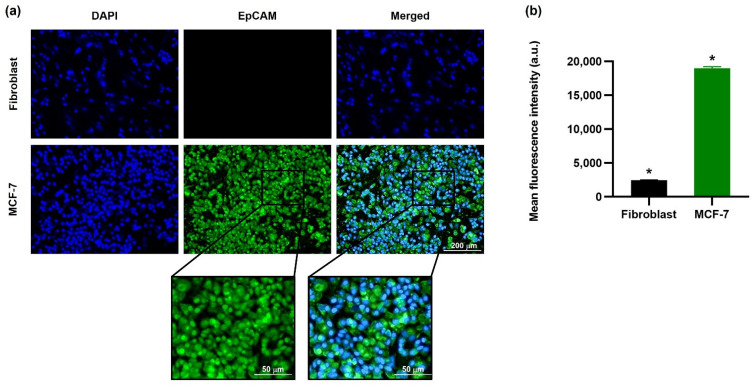
Co-localization of EINPs upon cellular binding/uptake. (**a**) EINPs were synthesized using PLGA cores, and their membranes were modified with anti-EpCAM labeled with FITC (green channel). DAPI was used to stain the nucleus (blue channel). Software was used to deconvolve all channels to remove out-of-focus fluorescent signals [10× images, scale bar = 200 μm]. (**b**) Quantification of the mean fluorescence intensities of EINPs on fibroblast and MCF-7 cells [bars show the mean ± SD (n = 3)]. * indicates a statistically significant difference between fibroblast and MCF-7 treated with EINPs (*p* < 0.05).

EpCAM protein is a potential target associated with breast-cancer cell proliferation, invasion, and metastases [25]. For this reason, the intracellular binding/uptake of both the MCF-7 and fibroblast cells was performed by fluorescent live cell imaging microscope analysis to verify MCF-7 cell EpCAM expression targeting efficiency so as to ascertain the quantity of surface immunofluorescence on the MCF-7 cell surface. The results (Figure 4a) illustrate that the MCF-7 cell membrane surface has a high green FITC compared to fibroblast cells. Furthermore, the mean fluorescent intensity shown in Figure 4b was confirmed by fluorescence spectrophotometer and established that the high green intensity of MCF-7 breast-cancer cells had a mean intensity of 18,990 a.u., and the fibroblast cells had a mean intensity of 2441 a.u. As a result, MCF-7 cell EpCAM overexpression is confirmed as a potential target for anti-EpCAM functionalized EINPs [26].

**Figure 5:**
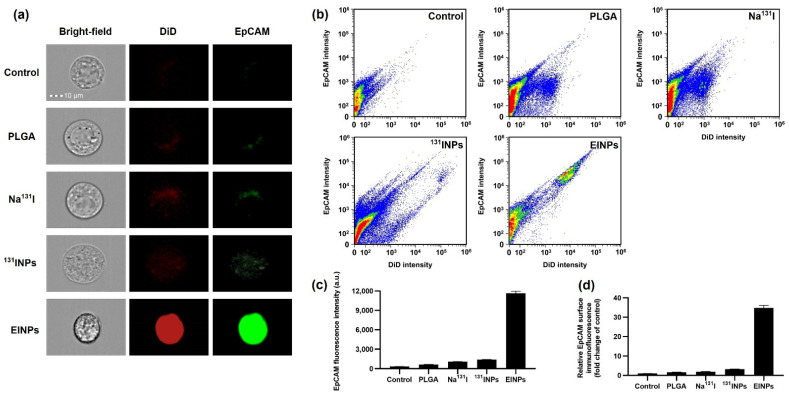
In vitro cell-surface immunofluorescence. (**a**) EINPs were fabricated with PLGA cores and their membranes modified using anti-EpCAM labeled with FITC (green channel) and DiD (red channel). The morphology of the cancer cell was imaged in bright-field (grey channel). All channels were imaged and fluorescent signals measured by flow cytometer [scale bar = 10 µm]. (**b**) The scatter plot of anti-EpCAM fluorescence intensities on MCF-7 cells treated with control, PLGA, Na^131^I, ^131^INPs, and EINPs on MCF-7 cells. (**c**) Quantification of mean fluorescence intensities of control, PLGA, Na^131^I, ^131^INPs, and EINPs on MCF-7 cells. (**d**) Relative EpCAM surface immunofluorescence (fold change of control) [bars represent mean ± SD (n = 3)].

Furthermore, to verify cellular binding/uptake, cell-surface immunofluorescence was performed. The bright-field and EpCAM (FITC) images of the control, PLGA, Na^131^I, ^131^INPs, and EINPs effects on MCF-7 cells were collected by flow cytometer to evaluate MCF-7 cell-surface immunofluorescence. Additionally, red fluorescence dye (DiD) was encapsulated within the PLGA, ^131^INPs, and EINPs and visualized, as shown in Figure 5a in under bright-field (grey channel), DiD (red channel), and EpCAM (FITC; green channel), demonstrating the distinct green fluorescent (FITC) dispersal patterns of the MCF-7-internalized Na^131^I, ^131^INPs, and EINPs. Similarly, the red fluorescent DiD confirms the specific cellular binding/uptake to MCF-7 cells. Moreover, the Na^131^I and ^131^INPs FITC concentrations were mainly limited to a few locations on the cell fringe, indicating that Na^131^I and ^131^INPs entered the MCF-7 cells due to receptor-mediated endocytosis. In comparison, the higher EINPs FITC concentrations indicate the green fluorescence was evenly distributed throughout the entire MCF-7 cytosol, in contrast to the lower concentrations of Na^131^I and ^131^INPs in a few locations on the cell periphery. This suggests that EINPs entered MCF-7 cells mainly by receptor-mediated membrane fusion and receptor-mediated endocytosis pathways. Furthermore, Figure 5b illustrates the DiD flow cytometry quantitative analysis of the control, PLGA, Na^131^I, ^131^INPs, and EINPs. It demonstrates that the anti-EpCAM functionalized EINPs binding specificity to MCF-7 cell receptors resulted in increased uptake of EINPs in comparison to the control, PLGA, Na^131^I, and ^131^INPs. Furthermore, the flow cytometry scatter plot displays the substantial shift in fluorescence on the targeted MCF-7 cells due to the anti-EpCAM labeled EINPs, suggesting specific binding between the anti-EpCAM conjugates and the MCF-7 target cells. In contrast, the control indicated minimal fluorescence shifting. In addition, the EpCAM mean fluorescent intensities (Figure 5c) illustrate the control mean fluorescent intensity as 335 a.u., against the EINPs mean fluorescent intensity of 11,663 a.u. Figure 5d illustrates the relative EpCAM surface immunofluorescence fold change compared to the control. The EINPs fold change was ~35-fold greater than the control.

### 3.4. Two-Dimensional (2D) In Vitro Live/Dead Cellular Binding/Uptake

**Figure 6:**
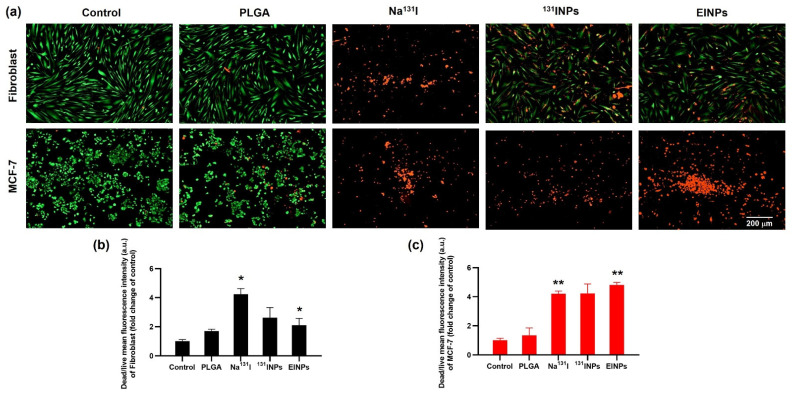
Two-Dimensional (2D) in vitro live/dead upon cellular binding/uptake (**a**) In vitro live/dead cell imaging of control, PLGA, Na^131^I, ^131^INPs, and EINPs. Fibroblast and MCF-7 cells were treated with PLGA at a fixed concentration of 10 mg/mL and Na^131^I, ^131^INPs, and EINPs activity 3.70 MBq for 72 h. Two-color fluorescence live (green channel) and dead (red channel) enabled the evaluation of live and dead cells to determine cell viability and cytotoxicity. Additionally, to eliminate out-of-focus fluorescent signals, all the channels were deconvolved by software [10× images, scale bar = 200 µm]. (**b**) Cytotoxicity quantification by dead/live mean fluorescence intensity of fibroblast (fold change of control) of control, PLGA, Na^131^I, ^131^INPs, and EINPs. (**c**) Cytotoxicity quantification by dead/live mean fluorescence intensity of MCF-7 (fold change of control) of control, PLGA, Na^131^I, ^131^INPs, and EINPs. [bars represent mean ± SD (n = 3)]. * the significance between Na^131^I and EINPs of fibroblast (*p* < 0.05). ** the significance between Na^131^I and EINPs of MCF-7 (*p* < 0.05).

A live/dead cell assay was performed to determine the MCF-7 cellular binding/uptake of EINPs (Figure 6) to verify that the EINPs anti-EpCAM functionalized nanotheranostic carriers accurately targeted the MCF-7 breast-cancer cells. The fibroblast and MCF-7 cells were treated by the control, PLGA, Na^131^I, ^131^INPs, and EINPs for 72 h. The results indicated by the live (green) channel and dead (red) channel enabled the assessment of live and dead cells. As shown in Figure 6a, the anti-EpCAM functionalized EINPs targeting MCF-7 had the most significant proportion of dead cells (red channel). In contrast, the control had very few visible dead cells. Furthermore, the EINPs treatment of the fibroblast cells indicated that the EINPs had limited cytotoxicity against fibroblast cells. In addition, the mean fluorescence intensity of the control, PLGA, Na^131^I, ^131^INPs, and EINPs was used to evaluate the mean fluorescent intensity per area of cytotoxicity (Figure 6b). The EINPs treatment of MCF-7 cells resulted in high green fluorescence intensity, thus suggesting that the anti-EpCAM targeting function of the EINPs precisely targeted MCF-7 EpCAM overexpression. The EINPs cellular binding/uptake to MCF-7 resulted in ~85% dead cells, compared to the Na^131^I treatment of MCF-7, which resulted in ~74 % dead cells. Furthermore, the assessment of cellular binding/uptake to fibroblast cells established that PLGA treatment resulted in ~45% dead cells, Na^131^I uptake resulted in 71.07%, ^131^INPs uptake resulted in ~28.48% dead cells, and EINPs resulted in ~35.28% dead cells. The cellular binding/uptake difference between fibroblasts treated by Na^131^I, ^131^INPs, and EINPs had a significance of *p* < 0.05. In summary, both the live/dead and mean fluorescent intensity determined that the anti-EpCAM targeting function of the EINPs had greater enhanced targeting and cellular binding/uptake to MCF-7 than Na^131^I and ^131^INPs.

### 3.5. Three-Dimensional (3D) Human Tumor Spheroid In Vitro Live/Dead Cell Imaging

**Figure 7:**
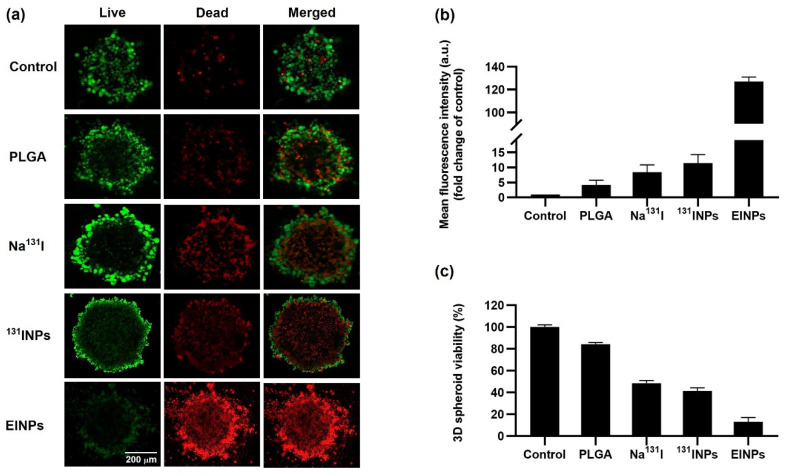
Three-dimensional (3D) human tumor spheroid in vitro live/dead cell imaging. (**a**) In vitro live/dead MCF-7 cell tumor spheroid imaging of control, PLGA, Na^131^I, ^131^INPs, and EINPs (I-131 activity of 3.70 MBq). Two-color fluorescence, live (green channel) and dead (red channel), enabled assessment of live and dead cells to evaluate cell viability. Scale bar = 200 µm. (**b**) Mean fluorescence intensity (fold change of control). (**c**) In vitro cytotoxicity study using CellTiter-Glo^®^ 3D cell assay of control, PLGA, Na^131^I, ^131^INPs, and EINPs against 3D human MCF-7 spheroids. Results represent mean ± SD (n = 3).

The three-dimensional (3D) human MCF-7 cell spheroid viability following EINPs treatment visualized by two-color fluorescence live/dead cell imaging is shown in Figure 7a. The MCF-7 spherical aggregates of ~525 µm in diameter were treated with control, PLGA, Na^131^I, ^131^INPs, and EINPs at an equivalent I-131 activity of 3.70 MBq (100 μCi) for 72 h. Performing two-color fluorescence, green (live) and red (dead), facilitated the assessment of 3D human MCF-7 cell spheroid live and dead cells. Furthermore, it was observed that spheroid size in all the treatment groups was similar, however visualization revealed a dissimilarity between the groups in the ratio of MCF-7 dead/live cells. In addition, the EINPs mean fluorescent intensity fold change compared to control was 127-fold higher (Figure 7b). The MCF-7 spheroid viability (Figure 7c) illustrates that EINPs treatment resulted in ~13% viability, ^131^INP ~41%, Na^131^I ~48%, and PLGA ~84%.

### 3.6. EINPs Penetration in Three-Dimensional (3D) Human Tumor Spheroid

**Figure 8:**
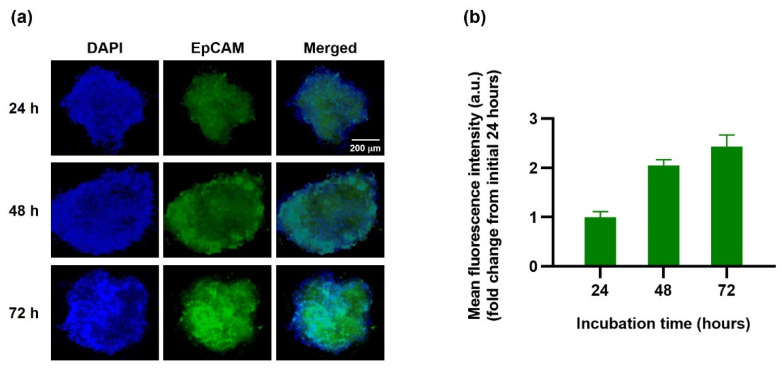
EINPs penetration in three-dimensional human (3D) MCF-7 tumor spheroid. (**a**) MCF-7 (EpCAM+) human spheroid fluorescence images treated with EINPs (green channel). The MCF-7 cell nucleus was stained with DAPI (blue channel). Scale bar = 200 µm. (**b**) Quantification of mean fluorescence intensity (fold change from initial 24 h) of EINPs penetration of MCF-7 spheroids with incubation times of 24, 48 and 72 h. Bars represent mean ± SD (n = 3).

The three-dimensional (3D) human tumor spheroid was subjected to in vitro penetration imaging to determine the EINPs cellular penetration in the 3D human MCF-7 spheroid model (Figure 8a). The fluorescence images of MCF-7 (EpCAM+) spheroids under EINPs treatment (green channel) were obtained at 24, 48, and 72 h. Moreover, the penetration results suggest the EINPs penetrated inside the 3D human MCF-7 spheroids increasingly over 72 h. As shown in Figure 8a, there was a stronger green signal inside the tumor spheroid as time progressed. In addition, (Figure 8b) the mean fluorescent intensity at 24 h was defined as 1-fold. At 48 h, the mean fluorescent intensity increased to 2-fold, and at 72 h, 2.5-fold. Hence, the results indicate that EINPs enhance nanoparticle penetration and uptake.

### 3.7. Analysis of Nanoparticle Hemolytic Properties In Vitro: Blood Compatibility

**Figure 9:**
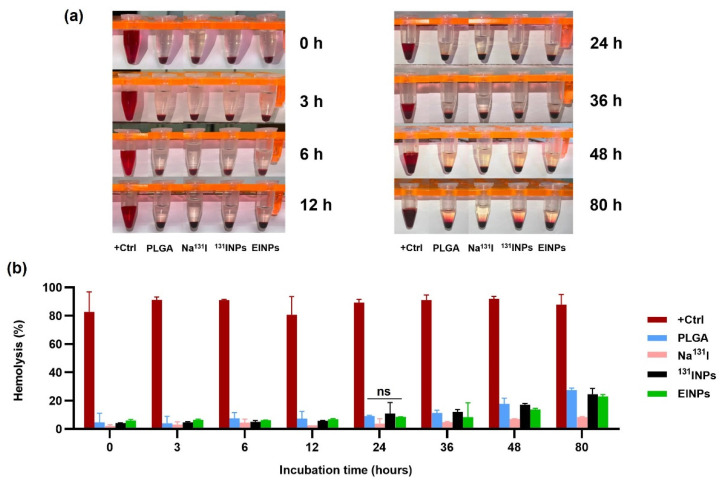
Analysis of nanoparticle hemolytic properties *in vitro*: blood compatibility was incubated with 8 × 10^9^ red blood cells/mL at 37 °C. (**a**) Red blood cells were pelleted down, and the supernatant was analyzed for lysed hemoglobin (DMSO as positive control), PLGA, Na^131^I, ^131^INPs, and EINPs after 0, 3, 6, 12, 24, 36, 48, and 80 h incubation. (**b**) Quantitative hemolytic interpretation of supernatant was analyzed for lysed hemoglobin. Data are given as mean ± SD (n = 3). Denote ns = no statistically significant difference in the hemolytic properties between PLGA, Na^131^I, ^131^INPs, and EINPs (*p* > 0.05).

Assessment of the in vitro hemolytic potential of the biomimetic anti-EpCAM functionalized EINPs to contribute to RBC damage was performed by hemolysis assay. DMSO was used as a positive control, and as anticipated, as shown in Figure 9a,b, the hemolysis at 0 h was 83%, increasing to 88% at 80 h. The hemolysis of Na^131^I was only 2%, increasing to 8% at 80 h, demonstrating that it caused minimal disruption to red blood cells, as an illustration of why it is commonly used in clinical applications. In addition, the hemolysis of Na^131^I at 0 h was 4%, increasing to 24% at 80 h. The EINPs hemolysis at 0 h was 6%, increasing to 23% at 80 h. However, the EINPs caused significantly less red blood cell destruction than the positive control, and the hemolysis of Na^131^I was not significantly different in comparison to the biomimetic anti-EpCAM functionalized EINPs (*p* = 0.117477), indicating that EINPs are biocompatible with blood and do not adversely affect red blood cells.

## 4. Discussion

Herein, our research further advanced biomimetic drug delivery platforms by coating NPs with human red blood cell membranes (hRBCm) and introducing Na^131^I, a radioactive theranostic agent and additionally an active targeting ligand, onto the hRBCm surface. Furthermore, we used human blood instead of murine blood to bring this study closer to clinical use. However, using hRBCm-NP in an in vivo mouse model is not possible because of immuno-incompatibility. Hence, we conducted this *in vitro* research with 3D human MCF-7 tumor spheroids instead of an in vivo mouse model. Research on novel biomimetic drug carriers is thriving, as the platform is expected to significantly influence the future treatment of challenging diseases. However, there has been limited research with hRBCm, and none to date combining hRBCm with a radioactive theranostic agent in a 3D model to treat breast cancer. Therefore, the authors designed this biomimetic drug delivery platform study considering its potential clinical translation by the pharmaceutical sector. Three-dimensional *in vitro* models are increasingly used to simulate cancer tumor microenvironments, as they are able to combine diverse patient-derived cells, which facilitates a greater knowledge of cellular interactions [27]. Increasingly, developments in the field of 3D models are providing more data for breast-cancer modeling, as well as for tumor-microenvironment stromal components, which promote the progression of cancers with therapeutic drug resistance [28]. Many properties of solid human tumors in vivo can be replicated in 3D multicellular spheroids, including their treatment resistance [29]. Furthermore, in this study, 3D multicellular spheroids were grown using the liquid overlay approach and ultra-low attachment plates, as they prevent MCF-7 cells from adhering to the surface of the well plate and stimulate MCF-7 cell aggregation and the creation of homogeneous floating spheroids. The MCF-7 human spheroids in this study were >500 µm in diameter and therefore able to mimic many characteristics of solid human tumors between 0.5 and 1 mm^3^ in volume [30].

Figure 1 illustrates how the construction of the targeted theranostic biomimetic drug delivery platform encompassing human red blood cell membrane-coated anti-EpCAM functionalized Na^131^I radiolabeled PLGA nanoparticles (EINPs) comprises three phases: (1) hRBCm-vesicle preparation, (2) PLGA NP synthesis and PLGA NP radiolabeling with Na^131^I, and (3) PLGA NP coating with hRBCm-vesicles and anti-EpCAM targeting ligand conjugation.

The concept of coating nanoparticles with biomimetic cell membrane to achieve stealth functionalization and enhance circulation time has gained considerable interest [31,32,33], as synthetic nanoparticles covered with a layer of natural cell membrane inherit unique and intrinsic properties of the original cell [34,35,36]. It has been reported that conventional PLGA NPs, after systemic administration, are removed from the bloodstream within 10 min, significantly impairing their therapeutic efficacy and targeting capability [37]. Additionally, the stability of membrane-coated nanocarriers is considerably enhanced due to their increased capacity to evade systemic clearance and macrophage uptake [38]. In addition, biomimetic nanocarriers can reduce drug leakage in physiological conditions [24]. Moreover, the therapeutic effect of nanomedicines is limited by their inability to penetrate solid tumors, as these formations have an irregular vasculature, high cellular density, dense extracellular matrix, and elevated osmotic pressure [39]. To overcome tumor hypoxia, Gao, Min, et al. designed a ~290 nm diameter RBCm-coated PLGA NP encapsulated with a perfluorocarbon, which was similar in size to the EINP ~295 nm. Their platform successfully delivered oxygen to reduce hypoxia by increasing the tumor-area oxygenation levels from 1.6% to 24% 24 h post-injection, enhancing radiotherapy treatment efficacy [40]. Furthermore, RBCm was used to coat metal-organic NP utilizing a TGF-β-blocking strategy, successfully reducing the extracellular matrix barrier and reducing hypoxia to enable nanocarrier penetration and overcome drug resistance [41]. Recently, a hemoglobin oxygen-carrier blood surrogate comprising a hemoglobin-loaded PLGA nanoparticle coated with nanozymes to prevent oxidation and coated with RBCm was shown to successfully preserve oxygen, and exhibited good antioxidant and stealth properties [42].

PLGA is a synthetic polymer approved for human therapy by the Food and Drug Administration (FDA) USA. Synthetic polymers have higher purity and greater reproducibility than natural polymers [43]. The PLGA NP is synthesized by the w/o/w double emulsion method to entrap the hydrophilic Na^131^I. As illustrated in Figure 2a, the PLGA NP has an initial z-average diameter of ~149 nm, which increases to ~264 nm after radiolabeling. In the same way, research by Zhang, Ruiguo, et al. identified that I-131 radiolabeling of mesoporous silica nanoparticles increased their size from 108 nm to 163 nm [44]. Furthermore, hRBCm coating resulted in an EINP z-average diameter of ~295 nm and a PDI of 0.297 (Figure 2b). These nanoparticles are acceptable for potential biomedical use [45].

The present-day bottom-up strategy using chemistry-centered conjugation methods to covalently conjugate membrane proteins with nanoparticles often results in protein denaturing and particle dimerization [46]. Hence, this study’s strategy was a top-down approach of translocating the hRBCm onto the NP surface and securing the membrane proteins, as confirmed in Figure 2e,f. The hRBCm glycan hydrophilic coating stabilizes the cells, and their sialyl residue negative charge affects hRBCm electrostatic interactions with PLGA NPs. While the bare PLGA NP surface had a charge of ~50 mV, after hRBCm coating, the EINP surface charge increased to −41 mV (Figure 2c). This increase in surface charge is a result of the less negatively charged hRBCm blocking the high negative charge of the carboxyl groups on the PLGA NP core surface [47]. Notably, Luk, Brian T., et al. evaluated RBCm-coated PLGA NP stabilization and verified that the RBCm polysaccharides played a vital role in the NP stability. Furthermore, in their study, the bare PLGA NP were ~100 nm diameter, with −45 mV zeta potential, close to the −41 mV zeta potential of the EINP of this study. The authors established that positively charged NP interaction with the RBCm electrostatic properties resulted in large polydisperse aggregates. In contrast, the negatively charged NPs resulted in a well-formed and consistent spherical NP core structure [48].

A radiolabeled nanoparticle contains a radionuclide in its stable form and can cause minor changes in the nanoparticle structure, such as the increase in EINP size after radiolabeling. Therefore, it is important to consider the effect of radiolabeling modifications on nanoparticle physicochemical characteristics, as radiolabeling should preserve the nanoparticle physicochemical characteristics, biodistribution and pharmacokinetics [49]. Furthermore, the Na^131^I is carrier-added to the PLGA core of the EINPs, and the high EINPs RCP implies the absence of other radioactive sources, which frequently occurs in nanomaterials due to vigorous construction purification protocols. Furthermore, the nanocarrier radiochemical purity must comply with purity standards set by the World Health Organization (WHO), which state that RCP must not be less than 95% [50]. Therefore, an unacceptable RCP could potentially cause radiopharmaceuticals to accumulate in a non-target organ and result in organ damage. The EINPs RCP was higher than the standard compared to the Na^131^I RCP, which declined below the purity standard (Figure 2g). Additionally, the radioactive encapsulation efficiency was over 95% (Figure 3b), similar to that found by Sakr, Tamer M., et al., who also used the non-chelator method to entrap I-131 in silver nanoparticles [51].

Furthermore, there are different strategies for nanocarrier radiolabeling or radiometal trapping, such as surface labeling, labeling surface-bound bioconjugates, and trapping at the aqueous phase during the synthesis of the nanoparticles. In this study, the double emulsion w/o/w method of nanoparticle construction was performed, and radioactive I-131 (Na^131^I) trapping was performed at the aqueous inner phase. Similarly, Li, Wei, et al. compared internal and external surface radiolabeling of nanoliposomes used to treat cervical cancer [52]. It was established that internal radiometal-trapped nanocarriers had better radiolabeling rates, delivered higher radioactivity doses than the external surface labeled nanocarriers, and achieved more significant cytotoxicity. Furthermore, the non-chelator method was utilized to incorporate Na^131^I directly into the PLGA NP core, obviating the requirement for external chelating agents. As a result, this approach is simpler and faster than chelator-based procedures—however, this depends on the type of nanomaterial and radionuclide utilized. Eliminating the use of a chelator often reduces the number of reaction steps, but more significantly, preserves the integrity of the nanomaterial, as the bulky chelator molecule may affect in vivo behavior. In addition, non-chelator-based solutions adapt a range of conventional radiolabeling reactions, while also using unique radiolabeling methods created particularly for radionuclide incorporation into nanomaterials [49,53]

Furthermore, when determining the quantity of radiolabeled Na^131^I to achieve cytotoxicity and correlating the amount of radiolabeled Na^131^I with the dosage to be dispensed, the specific activity needs to be considered. This is because the higher the specific activity, the less dosage is required to achieve the same activity level, which is critical for imaging investigations and therapeutic applications in which the quantity of activity is proportional to therapeutic efficacy. A high specific activity corresponds to sufficient activity at lower concentrations, enabling microdosing clinical investigations. The FDA strongly recommends the pre-approval of novel medications that have low-risk profiles [54]. Additionally, Na^131^I is a recognized treatment for cancer tumors as it has a small linear energy transfer (LET) of around 0.25 keV/µm, and the electron radiation energy is absorbed local to the radiation. Moreover, the Na^131^I emission of high-energy beta and gamma rays functions as an internal radiotherapeutic, as the ionizing radiation damages cancer cell DNA [55]. Initial DNA damage can occur as a consequence of the ionizing particle’s direct contact with the DNA via energy depositions through ionization and excitation, and also as an indirect result of the interactions of free radicals created by radioactive particles ionizing the water around the cells which accounts for 70% of DNA damage [56]. Furthermore, there are two processes by which I-131 radiation inhibits cells. One is cell reproductive failure induced by radiation, and the other is apoptosis (programmed cell death).

Additionally, there have been a number of methods developed for attaching bioactive moieties to nanoparticles. These are categorized as follows: biotin-streptavidin conjugation, covalent coupling, ligand-receptor conjugation, lipid fusion, and adsorption. To amalgamate the hRBCm vesicle coating and anti-EpCAM targeting ligand to the PLGA NP surface, it is initially necessary to coat the hRBCm onto the PLGA NP. In this study, the surface of the anti-EpCAM functionalized hRBCm-nanoparticles was synthesized by the lipid-insertion method. Biotin-functionalized lipids were incorporated into the hRBCm vesicles during the extrusion process and readily linked to the free streptavidin-FITC, with biotin-functionalized anti-EpCAM then binding to the streptavidin on the membrane, as shown in Figure 1. As a result, we calculated ~33 anti-EpCAM molecules per 1 PLGA nanoparticle, as reported previously [57,58].

In addition, streptavidin affinity to biotin results in a powerful noncovalent biological interaction. A single streptavidin monomer binding with one biotin molecule results in a streptavidin protein binding with four biotins with excellent specificity and affinity (K_d_ ~10^−14^ mol/L). Some additional advantages are that it is highly specific, has a rapid rate, is resistant to temperature and pH changes, and can withstand denaturing agents and organic solvents. Additionally, biotin-based conjugates are easily synthesized and also have a more negligible effect on the biomolecules [59]. hRBCm was mixed with DSPE-PEG-biotin, and the biotin lipid chains became interleaved with the hRBCm lipid bilayer. Furthermore, the biotinylated anti-EpCAM RBC was incubated with streptavidin-fluorescein isothiocyanate (FITC) to create binding sites to conjugate the EpCAM antibodies targeting the MCF-7 cells with the hRBCm. In addition, the streptavidin-FITC enables the detection of the biotinylated EpCAM antibody of MCF-7 cellular binding/uptake. Notably, the anti-EpCAM ligand provides local stimuli, ensuring only the targeted MCF-7 cells will be stimulated [60]. 

Moreover, this study employed Na^131^I in combination with anti-EpCAM to specifically target NIS and EpCAM in MCF-7. Thus, we utilized a Na^131^I-labeled nanocarrier loaded with anti-EpCAM to specifically target NIS and overexpression of CD326 in MCF-7 [61,62]. For a number of decades, iodide transport in thyroid cells through NIS has been the basis for using radioiodine to successfully diagnose and treat differentiated thyroid carcinoma [63], because NIS is essential for the absorption of iodide, which is necessary for thyroid hormone production. However, it is also expressed in non-thyroidal tissues, such as the lactating mammary gland in human breast tumors. For instance, Cline, Benjamin L., et al. developed a potassium iodide (KI) NP that utilized NIS targeting to deliver iodine and radiosensitization [64]. The results indicated a substantial enhancement of breast-cancer tumor suppression, increased nanoparticle circulation time, controlled iodide release, and NIS-mediated cellular binding/uptake.

In addition, the efficacy of nanoparticle cellular targeting, uptake, and intracellular trafficking can be enhanced by modifying their physicochemical properties, such as shape, size, and surface properties [65,66]. For this reason, nanoparticle characterization has an essential role in the development of nanocarriers. Nanoparticle particle size and PDI are the major physicochemical characteristics affecting endocytosis cellular binding/uptake. Hence, the physicochemical properties of the EINPs were verified and illustrated in Figure 2. The results indicated that after radiolabeling, the PLGA NPs increased in size from ~149 nm to ~265 nm prior to hRBCm coating, and after hRBCm coating, the EINPs increased to ~295 nm. In the case of nanocarriers, drug administration efficacy depends on the nanoparticle diameter, PDI, and zeta potential. For nanoparticle sizes ranging from 200 nm to 2000 nm, administration is intravenous or intramuscular [67]. The EINP PDI is 0.297 (Figure 2b). Nanocarriers with PDI of 0.3 or below are considered acceptable, which means that EINP nanoparticles are homogenous nanoparticles [45]. Furthermore, the EINP zeta potential of −41.1 mV (Figure 2c) indicates the nanoparticles had good particle stability. Zeta potential is indicative of stability in colloidal solutions. For example, a charged particle with a surface potential greater than +40 mV or less than −40 mV is considered to have good particle stability in colloidal solutions [68]. The size and zeta potential of the EINPs in this study suggest that the hRBCm coating was satisfactory, as verified by transmission electron microscopy (Figure 2d), which shows the hRBCm layer surrounding the NP core. Additionally, the retention of the hRBCm protein on the EINPs surface was verified by the sodium dodecyl sulfate-polyacrylamide gel electrophoresis (SDS-PAGE) protein analysis (Figure 2e) and Western blotting (Figure 2f), indicating that compared to the hRBCm vesicles, the EINPs preserved the majority of the proteins. This confirmed that the CD47 glycoproteins successfully translocated to the EINPs.

Additionally, the MCF-7 cell viability determined that the anti-EpCAM functionalized EINPs had more significant toxicity and illustrated enhanced cytotoxicity targeting (Figure 3c). Furthermore, the initiation of nanoparticle cytotoxicity is governed by its cellular entry path and intracellular nanoparticle localization. Therefore, identifying nanoparticle intracellular trafficking and their cellular binding/uptake is essential in designing a safe and efficient nanotherapeutic [69]. The in vitro targeting efficiency confirmed the ability of EINPs to target NIS and EpCAM expression of MCF-7 cells (Figure 4). This illustrates why there is scarcely any fluorescence on the fibroblast cells, and a significantly higher intensity on the MCF-7 cells, which had a mean fluorescence intensity 7.8-fold higher.

Furthermore, MCF-7 cellular binding/uptake of EINPs further verified that the anti-EpCAM functionalized biomimetic nanotheranostic accurately targets MCF-7 cells, enhancing their intracellular delivery and cytosolic release (Figure 5). Moreover, MCF-7 in vitro uptake of the EINPs was evenly distributed throughout the MCF-7, as confirmed by the higher FITC (EpCAM) and DiD concentrations (Figure 5a). It was also determined that the anti-EpCAM-FITC binding specificity to MCF-7 cell receptors resulted in increased uptake of EINPs (Figure 5c,d). Furthermore, EINPs uptake was confirmed by a substantially higher mean fluorescent intensity than the non-targeted ^131^INPs, signifying more than a 10-fold increase. Additionally, the in vitro 2D live/dead assay upon cellular binding/uptake confirmed the anti-EpCAM targeting ability of the EINPs, which indicated its enhanced targeting and cellular binding/uptake in comparison to Na^131^I and ^131^INPs (Figure 6). Further confirmation of the EINPs cytotoxicity in the 3D human MCF-7 spheroid model achieving a MCF-7 viability of ~13% is shown in Figure 7. Furthermore, EINPs penetration into the >500 µm diameter 3D MCF-7 tumor spheroid increased over 72 h (Figure 8).

The hemolytic capabilities of nanoparticles are a frequently used parameter in the research of nanoparticle interaction with blood components. The induced hemolysis was calculated by comparing the observed hemoglobin concentration to the total hemoglobin, and verified that EINPs are biocompatible with blood and do not adversely affect RBCs. Additionally, the hemolytic properties of the EINPs were determined as having no adverse effect on RBCs (Figure 9).

## 5. Conclusions

Polymeric NPs coated with hRBCm constitute a novel biomimetic nanocarrier with a Na^131^I cargo, and an anti-EpCAM targeting nanotheranostic for breast cancer was developed. This study demonstrated the excellent ability of EINPs to target EpCAM-overexpressed breast-cancer cells in comparison to the non-targeted NPs, resulting in greater cellular binding/uptake. The Na^131^I was incorporated directly into the PLGA NP core by the non-chelator method to ensure retention of the integrity of the nanomaterial. The hRBCm biomimetic coating is known to provide increased stability and reduce payload leakage in physiological conditions. NIS plays a crucial role in enhancing Na^131^I delivery, and was utilized in conjunction with anti-EpCAM to target breast-cancer cells specifically.

We demonstrated that the optimized EINPs biomimetic nanotheranostic enhanced cytotoxicity at 72 h with three-fold higher cytotoxicity than the ^131^INPs and Na^131^I. Furthermore, the anti-EpCAM targeting function of the EINPs showed enhanced targeting ability and cellular binding/uptake to MCF-7. The MCF-7 fluorescence intensity with EINPs was 8.24-fold higher than with Na^131^I, and 10.74-fold higher than the non-targeted non-hRBCm-coated ^131^INPs. Additionally, the EINPs had a favorable safety profile, as determined by hemolysis.

In this study, we verified that our targeted hRBCm biomimetic nanotheranostic, the hRBCm-coated PLGA nanocarrier, successfully accomplished the delivery of a radiotherapeutic agent. Therefore, we believe this clinically translatable biomimetic nanotheranostic delivery platform has the potential to be utilized for the delivery of radiotherapeutics for the treatment of other forms of cancer in the future.

## Data Availability

The data presented in this study are available on request to the corresponding author.
